# Risk factors for complications from challenging lower third molar extractions in tertiary hospital patients

**DOI:** 10.2340/aos.v83.42464

**Published:** 2024-12-18

**Authors:** Sanna J. Koskela, Irja Ventä, Johanna Snäll, Hanna Välimaa, Miika Toivari

**Affiliations:** a Department of Oral and Maxillofacial Diseases, University of Helsinki, Helsinki, Finland; b Department of Oral and Maxillofacial Diseases, Helsinki University Hospital, Helsinki, Finland; c Meilahti Vaccine Research Center MeVac, Department of Infectious Diseases, University of Helsinki and Helsinki University Hospital, Helsinki, Finland

**Keywords:** Complications, tooth extraction, risk factors, third molar, tertiary health care

## Abstract

**Objective:**

Third molar extraction is a common procedure with occasional complications. This study aimed to determine the incidence and types of complications in challenging lower third molar extractions and to identify complication risk factors in tertiary hospital patients.

**Material and methods:**

A retrospective cohort study was conducted on 354 patients who underwent unilateral lower third molar extraction during a 2-year period in 2018–2019 at Helsinki University Hospital. The outcome was the presence of a complication, and patient-related and operation-related variables served as determinants. Statistical analyses included Mann–Whitney U and Chi-squared tests, and binary logistic regression.

**Results:**

Complications occurred in 16.7% of patients. The most common complication was local infection (7.6%), followed by nerve injury (5.6%). The complication risk was 3.7-fold (95% confidence interval (CI) 1.97–6.77, *p* < 0.001) higher in extractions defined as demanding than in routine operative extraction. If the third molar was acutely infected, the complication risk increased 2.0-fold (95% CI 1.08–3.75, *p* = 0.027).

**Conclusions:**

Due to the high rate of complications in challenging extractions, scheduling a follow-up visit is important, and risk factors must be considered properly, especially in demanding extractions and in acutely infected third molars.

## Introduction

Third molar extraction is a common procedure with occasional complications. Most complications are minor, although there is a risk of unfavorable outcomes and permanent harm, particularly in challenging extractions with unusual pathology. In addition to challenging extractions, other typical reasons for third molar treatment in a tertiary hospital comprise the patient’s severe medical condition and acute local or deep-space odontogenic infection [1]. However, the complication profile of challenging lower third molar extractions of hospital patients remains to be elucidated.

Complications in lower third molar surgery are well known and include the usual postoperative morbidities [2, 3], infections [4, 5], mandibular fractures [6], and nerve injuries [7, 8]. To reduce the risk of complications, several studies have identified such risk factors as increasing age [9], preoperative third molar symptoms [10], and surgical complexity [11]. However, studies focusing only on challenging extractions in hospital settings are rare. Since the mandibular third molar is the most commonly extracted tooth associated with malpractice claims [12], it is appropriate to also clarify the risk factors for complications in hospital patients.

The aim of this study was to investigate the incidence and types of complications in challenging lower third molar extractions in tertiary hospital patients. The specific aim was to identify risk factors for complications. We hypothesized that the complications are the same as in earlier studies but the occurrence rates differ.

## Materials and methods

### Study design

The study was designed to examine retrospectively the complications occurring in challenging third molar extractions in tertiary hospital patients. Data on all inpatients and outpatients with challenging mandibular third molar extractions visiting the Department of Oral and Maxillofacial Surgery at Helsinki University Hospital from 1 January 2018 to 31 December 2019 were retrieved from the electronic patient register according to the treatment code of extraction. Records on extraction visits during the 2-year period and follow-up visits during a 3-year period up to 31 December 2020 were examined.

The extraction difficulty was determined by a treatment code [13] recorded in the electronic patient register. All treatment codes for operative extractions were sampled and for non-operative extractions the one with the label ‘demanding’ was sampled. The three codes included were as follows: demanding non-operative extraction with separation but not raising a mucoperiosteal flap (code EBA05), routine operative extraction with osteotomy and separation (code EBA10), and demanding operative extraction (code EBA12).

To avoid possible statistical bias, patients with bilateral challenging extractions were excluded. The missing data analysis showed that excluded patients (*n* = 95) were younger than included patients (*p* = 0.006), while sex (*p* = 0.501) and number of complications (*p* = 0.651) did not differ between the two groups.

### Study variables

The primary outcome variable was the recorded intra- or post-operative complication, classified as present or absent. The secondary outcome variable was complication type: (1) infection (formation of pus, bone sequester, alveolar osteitis, or osteomyelitis), (2) neurosensory disturbance, (3) prolonged pain requiring extended postoperative control, (4) retained root fragment in the extraction socket or in soft tissue, and (5) other rare complications.

Patient-related explanatory variables were age, sex, medical condition, regular use of medication, medical problem increasing infection risk, smoking, and anxiety about dental treatment. Medical conditions comprised heart disease, arterial hypertension, diabetes, respiratory disease, and bleeding disorder. Medical problem that increase infection risk included diseases and treatments, e.g. diabetes, dialysis, and immunosuppressive medication or condition.

Operation-related explanatory variables comprised the following: method of extraction, side of surgery, acuteness of extraction, primary cause of treatment in tertiary health care, unit of extraction (elective or emergency unit), type of anesthesia, use of antibiotics, and operating craniomaxillofacial surgeon (trainee, consultant, or both). The acuteness of third molar extraction was grouped as non-acute odontogenic indication or acute odontogenic infection (pain, swelling, pus, fistula, abscess, or pericoronitis with symptoms). Primary cause of treatment in tertiary health care was categorized as complex dentoalveolar pathology, patient`s state requiring hospital facilities, or acute odontogenic infection. Use of antibiotics was classified as no antibiotics, preoperative single high-dose prophylaxis only, single-dose prophylaxis together with postoperative antibiotics for 3 days or more, and postoperative antibiotics without single-dose prophylaxis.

### Statistical analysis

The observational unit was a patient. Differences between continuous variables were analyzed using the Mann–Whitney U test and categorical variables using Chi-squared test. A *p* value < 0.05 was considered significant. Statistical analyses were conducted using IBM SPSS Statistics version 29 (IBM Corp., Armonk, NY, USA).

Descriptive statistics of patient-related and operation-related variables were tabulated. All explanatory variables were cross-tabulated according to presence or absence of complications, and all comparisons with a statistically significant difference were reported in a table. Binary logistic regression analysis was used to ascertain the effect of explanatory variables on the likelihood of a complication. Odds ratios (ORs) and their 95% confidence intervals (CIs) are presented. The Hosmer and Lemeshow test was used to evaluate the goodness of fit of the model.

### Ethical considerations

This study followed the Declaration of Helsinki on medical research protocols and ethics. The Institutional Review Board of the Head and Neck Center, Helsinki University Hospital approved the study (HUS/126/2021). No informed consent from patients was required due to the retrospective study design. For data protection reasons, results are not presented if the frequencies were less than five, and therefore, some subgroup combinations in the analyses were made.

## Results

A total of 354 patients (55% men, *n* = 195 and 45% women, *n* = 159) were included in the study. The mean age of patients was 38.7 years (range 13–99 years). Descriptive statistics of patient-related variables are presented in Table 1.

**Table 1:** Characteristics of the 354 patients with lower third molar surgery.

Characteristic	*n*	%
Age (years)		
Mean 38.7; median 33.0; range 13–99		
Sex		
Men	195	55.1
Women	159	44.9
Medical condition		
Diagnosed disease	217	61.3
No diagnosis	137	38.7
Regular medication		
Yes	187	52.8
No	167	47.2
Medical problem increasing infection risk		
Present	77	21.8
Absent	277	78.2
Smoking		
Yes	69	19.5
No	218	61.6
No information	67	18.9
Anxiety about dental treatment		
Yes	13	3.7
No	341	96.3

Descriptive statistics of operation-related variables are presented in Table 2. Most patients (92.4%) underwent operative extraction, and general anesthesia was used in 22.6% of all extractions.

**Table 2:** Characteristics of operation-related variables in 354 patients.

Characteristic	*n*	%
Method of extraction		
Demanding non-operative	27	7.6
Routine operative	229	64.7
Demanding operative	98	27.7
Side of surgery		
Right mandible	180	50.8
Left mandible	174	49.2
Acuteness of third molar extraction		
Acute infection	93	26.3
Non-acute odontogenic indication	261	73.7
Primary cause of treatment in tertiary health care		
Complex dentoalveolar pathology	82	23.1
State requiring hospital facilities	179	50.6
Acute infection	93	26.3
Unit of extraction		
Elective unit	320	90.4
Emergency unit	34	9.6
Anesthesia		
Local	229	64.7
Local and sedation	45	12.7
General	80	22.6
Use of antibiotic		
No	13	3.7
Preoperative single-dose	64	18.1
Single-dose and postoperative for 3 days or more	271	76.5
Postoperative without single-dose of prophylaxis	6	1.7
Operating craniomaxillofacial surgeon		
Resident	298	84.2
Senior	7	2.0
Resident and senior	49	13.8

**Figure 1:**
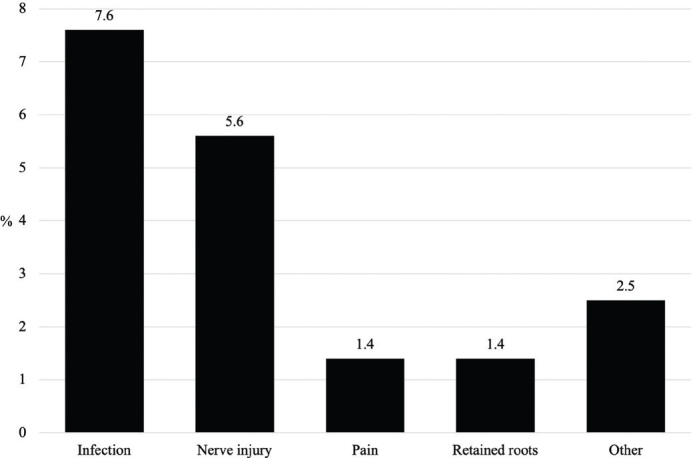
Distribution of complications (*n* = 66) in challenging lower third molar extractions of 354 hospital patients. Each patient may have had one or more complications.

A total of 66 intra- or post-operative complications occurred in 59 patients (16.7%). The most common complication was local infection, followed by neurosensory disturbance (Figure 1). The inferior alveolar nerve (IAN) was severed in 4.5% of all patients and the remainder were lingual nerve injuries. The follow-up revealed that the neurosensory deficiency was transient in 2.5% of all patients, 1.7% of cases were permanent within the limits of the follow-up, and 1.4% of patients were not seen after the first control visit to determine the duration of the deficiency. Seven patients sustained two complications, mostly infection and neurosensory disturbance together. Re-operation such as surgical revision or drainage of an abscess was needed in 23 patients (6.5% of all patients).

Bivariate analysis of explanatory variables and the occurrence of complications showed that the mean age of patients was higher in the complication group than in the non-complication group (Table 3). Other significant factors associated with complications were presence of regular medication, extractions labelled as demanding, existing acute odontogenic infection of the third molar, acute odontogenic infection as the cause of referral to hospital, and general anesthesia.

**Table 3:** Comparison of explanatory variables according to presence or absence of postoperative complication in third molar surgery in 354 patients.

Variable	Complication				*P*
	Yes		No		
	*n* = 59	%	*n* = 295	%	
Age (years)					0.002^a^
Range	20–86		13–99		
Mean	43.9		37.6		
Regular medication					0.025^b^
Yes	39	20.9	148	79.1	
No	20	12.0	147	88.0	
Method of extraction					< 0.001^b^
Demanding extraction (non-operative/operative)	38	30.4	87	69.6	
Routine operative	21	9.2	208	90.8	
Acuteness of third molar extraction					0.002^b^
Acute infection	25	26.9	68	73.1	
Non-acute odontogenic indication	34	13.0	227	87.0	
Primary cause of treatment in tertiary health care					< 0.001^b^
Complex dentoalveolar pathology	19	23.2	63	76.8	
State requiring hospital facilities	15	8.4	164	91.6	
Acute infection	25	26.9	68	73.1	
Anesthesia					< 0.001^b^
Local	24	10.5	205	89.5	
Local and sedation	12	26.7	33	73.3	
General	23	28.7	57	71.3	

Only explanatory variables with statistical significance are presented.

a Mann–Whitney U;

b Chi-squared.

In logistic regression analysis, two of the predictor variables emerged as predicting complications (Table 4). In demanding extractions, complication risk was 3.7-fold (95% CI 1.97–6.77) higher than in routine operative extractions. Acute third molar infection increased the complication risk by 2.0-fold (95% CI 1.08–3.75) compared with non-acute odontogenic indication. In this model, age and sex did not have a significant effect on complications. The regression model predicted 84.7% of cases correctly. The Hosmer and Lemeshow test indicated excellent fit (*p* = 0.996) of the model.

**Table 4:** Adjusted binary logistic regression analysis predicting the likelihood of complication in third molar surgery in 354 patients.

Category	OR (95% CI)	*P*
Age	1.0 (0.99–1.03)	0.164
Sex [women vs. men]	1.8 (0.97–3.24)	0.063
Type of extraction [demanding vs. routine]	3.7 (1.97–6.77)	< 0.001
Acuteness of extraction [acute infection vs. non-acute]	2.0 (1.08–3.75)	0.027

Reference group information in square brackets.

OR = Odds ratio; CI = Confidence interval.

## Discussion

This study examined complications and their risk factors in challenging lower third molar extractions in tertiary hospital patients. The findings showed that the complication rate was high. Several risk factors were identified; the most powerful of these were extraction defined as demanding and acutely infected third molar.

The complication rate (16.7%) is high, ranking between previously reported rates of 2.4% and 22.0% [14–16]. The highest complication rate was reported in a German study on hospital patients in which 22% of elderly patients (> 65 years) and only 3.1% of younger patients sustained complications [16]. The lowest complication rates were reported in ambulatory studies (9.8%) or in extractions with local anesthesia or sedation (2.4%) [14, 15]. However, comparison of complication rates between the present and earlier studies is difficult due to differences in study designs, numbers of third molars extracted per patient, and methods of extraction.

The most common complication was a local infection (7.6%). A higher rate (22.1%) than this was reported in the German hospital study on extracted third molars of varying difficulty levels [11]. In that study, antibiotics were not generally used. A lower rate than the present finding was reported in a Brazilian hospital study, at 3.8% of extracted teeth in healthy patients [17]. The lowest infection complication rate was reported in a Spanish outpatient study, where delayed-onset infections occurred in 1.5% of lower third molar extractions [18]. Once again, comparison with earlier studies is hindered by differences in study designs and numbers of extracted teeth and whether the unit of observation was a tooth or a patient.

The second most common complication in our study was a neurosensory disturbance (5.6%), which is higher than in previous studies (1.2% – 5.5%) [19, 20]. The present rate of temporary nerve disturbance (2.5%) falls within the limits of earlier studies (0.9% – 3.0%) [19, 21]. However, the design of nerve injury studies varies and tooth-associated cysts are usually excluded due to increasing complication risk [22]. In our study, one-third of the third molars with complications had some type of dentoalveolar pathology, including several cysts. Also, most complications occurred under general anesthesia. Choosing general anesthesia suggests that the tooth anatomy and the localization of IAN had been too challenging to be performed under local anesthesia. Therefore, as patient co-operation was lacking under general anesthesia, nerve damage may have occurred more easily.

The most prominent risk factor for complication was a demanding extraction, which increased the risk by 3.7-fold. Similar findings were reported in earlier studies using logistic regression analysis. In the Belgian multi-center study, intraoperative osteotomy was a risk factor for a higher occurrence of pain, trismus, swelling, and altered lower lip sensation [2]. A Spanish hospital study on third molar extractions showed that an easy surgery has 81.4% lower risk of complications than a difficult surgery [23].

Another significant risk factor in this study was an acute infection of a third molar, which increased the complication risk by 2.0-fold. Similarly, the recent European multi-center study reported a correlation between preoperative symptoms of mandibular third molar and postoperative pain (risk ratio 3.6) and facial swelling (risk ratio 2.1) [10]. Likewise, a Japanese study showed using multivariate logistic regression that preoperative mandibular third molar infection increased post-extraction infection complication by 4.68-fold [24]. A slightly different outcome was reported in the US research using extended statistical methods, where third molar infection was not a significant factor, but the presence of periodontal condition increased the risk of complications by 1.09-fold [5].

This study’s strengths were that a multitude of explanatory variables were included in the analysis and extractions were performed in both elective and emergency units. Therefore, the findings can be generalized across hospital patients with challenging mandibular third molar extraction. Contrary to many earlier studies, the present analysis focused only on unilateral extractions, instead of bilateral or bimaxillary extractions, to avoid possible statistical bias. When only one tooth is extracted, the complications can be attributed exactly to that tooth. The study’s retrospective nature set some limitations. Extractions were performed by several clinicians. In addition, the procedures were performed in the training hospital of the specialization training. It can be assumed that the trainee’s experience also varied, but it is impossible to find exact information about the trainee’s expertise, so this was not assessed in more detail. Also, a treatment code indicating the difficulty level of the extraction was recorded in the register by the clinician’s judgement. While the Finnish Institute for Health and Welfare instructs the usage of the treatment codes [13], variations in usage likely exist. Therefore, when gathering the data from patient records, compatibility of the procedure and the treatment code were checked. The follow-up visits were restricted to a 3-year period, and thus, evaluation of the permanence of nerve injuries was not complete. Further studies should focus on tooth-related and anatomical factors in the prevention of complications.

In conclusion, complication occurrence in third molar extractions depends on a variety of predictors. Relative to previous ambulatory studies, the complication rate in challenging extractions of mandibular third molars in hospital patients here was higher. Local infection was the most common complication, consistent with earlier research, and the incidence of nerve injuries was higher than in previous studies. The profile of a hospital patient sustaining a complication is an older person with regular medication having a surgically complex extraction of an acute mandibular third molar under general anesthesia. A follow-up visit is therefore important to schedule, and the risk factors, especially a demanding extraction and an acutely infected third molar, must be considered in treatment.
